# Clinical and Microbiological Effects of *Streptococcus salivarius* K12 Lozenges and Zinc Mouthrinse on Persistent Intra-Oral Halitosis

**DOI:** 10.3390/microorganisms14050990

**Published:** 2026-04-28

**Authors:** Adrian Bolos, Otilia Cornelia Bolos, Edida Maghet, Alexandra Ioana Danila, Raluca Briceag, Bogdan Andrei Bumbu

**Affiliations:** 1 Department of Oral Rehabilitation, Faculty of Dental Medicine, Specialization of Dental Technology, “Victor Babes” University of Medicine and Pharmacy Timisoara, 300041 Timisoara, Romania; bolos.adrian@umft.ro; 2 Department of Dental Aesthetics, Faculty of Dental Medicine, “Victor Babes” University of Medicine and Pharmacy Timisoara, 300041 Timisoara, Romania; bolos.otilia@umft.ro (O.C.B.); edida.maghet@umft.ro (E.M.); 3 Department of Anatomy and Embryology, “Victor Babes” University of Medicine and Pharmacy Timisoara, 300041 Timisoara, Romania; 4 Faculty of Dental Medicine, Ovidius University of Constanta, 7 Ilarie Voronca Street, 900684 Constanta, Romania; 5 Department of Dental Medicine, Faculty of Medicine and Pharmacy, University of Oradea, 410073 Oradea, Romania; bogdanbumbu@uoradea.ro

**Keywords:** halitosis, mouthwashes, zinc compounds, probiotics, quality of life

## Abstract

Background and Objectives: Halitosis is a common condition with substantial psychosocial impact, frequently driven by intra-oral biofilm, tongue coating, and reduced salivary clearance. This study compared the short-term effectiveness of standardized counseling alone, probiotic lozenges containing *Streptococcus salivarius* K12, and a zinc-containing mouthrinse in adults with persistent intra-oral halitosis. Materials and Methods: In this 4-week, parallel-group, randomized pragmatic trial, 117 adults with bothersome halitosis for at least 3 months and baseline organoleptic score ≥ 2 were allocated 1:1:1 to standard care, probiotic lozenges, or zinc mouthrinse. All participants received standardized counseling and tongue cleaning instructions. The primary endpoint was change in volatile sulfur compounds (VSCs) measured by portable sulfide monitoring. Secondary outcomes included organoleptic score, Halitosis Associated Life-Quality Test (HALT), Oral Health Impact Profile-14 (OHIP-14), tongue coating, plaque, and salivary *Solobacterium moorei* quantified by qPCR. Results: Baseline demographic, clinical, and biochemical characteristics were comparable across groups. All interventions improved outcomes over 4 weeks, but improvements followed a consistent gradient favoring zinc mouthrinse, followed by probiotic lozenges, then standard care. Mean VSC reduction was −12.7 ± 33.9 ppb with standard care, −47.3 ± 42.2 ppb with probiotics, and −78.5 ± 36.3 ppb with zinc mouthrinse (*p* < 0.001). Organoleptic scores improved by −0.2 ± 0.7, −0.8 ± 0.8, and −1.2 ± 0.8, respectively (*p* < 0.001). HALT and OHIP-14 scores showed parallel reductions, and moderate/severe halitosis at week 4 remained most frequent in the standard care group (58.9%) and least frequent in the zinc group (20.5%; *p* = 0.004). Conclusions: Both active adjunctive strategies improved intra-oral halitosis beyond standardized counseling alone, but the zinc-containing mouthrinse produced the greatest short-term benefits across objective, clinician-rated, and patient-reported outcomes. These findings support zinc-based rinses as a practical short-term adjunct for managing persistent intra-oral halitosis in outpatient dental care. Durability after discontinuation and potential relapse beyond 4 weeks were not assessed in this trial.

## 1. Introduction

Halitosis is a common, patient-relevant complaint with disproportionate psychosocial consequences, ranging from embarrassment and social avoidance to reduced professional confidence and perceived quality of life. Population estimates vary because of differences in definitions and measurement methods, but clinically relevant halitosis is generally reported in a sizable minority of adults, while transient “morning breath” is even more frequent. This burden makes halitosis an important target for pragmatic interventions that can be implemented in routine dental care without complex infrastructure [1,2].

Most cases are intra-oral in origin (often cited as 85–90%), with malodor primarily driven by microbial proteolysis of proteins and peptides into volatile sulfur compounds (VSCs), particularly hydrogen sulfide and methyl mercaptan. The posterior tongue dorsum is a key ecological niche because its papillary surface retains desquamated cells and food debris, supporting anaerobic biofilms with high odorant-generating potential. Periodontal inflammation and deeper pockets can amplify these pathways by increasing bacterial biomass and providing protein-rich substrates, while a smaller but clinically important subset of patients have extra-oral contributors that warrant targeted screening when oral causes do not explain symptoms or treatment response [1,2,3].

Saliva modulates halitosis risk through mechanical clearance, buffering, and antimicrobial functions. Importantly, “dry mouth” symptoms do not always align with objective flow measures, so studies that assess both perceived xerostomia and measured salivary flow can better characterize clinically meaningful phenotypes. In a clinical cohort measured by gas chromatography, extremely low resting salivary flow was associated with higher VSC concentrations and greater tongue coating burden, and, together with increased tongue coating and probing pocket depth > 4 mm, emerged as a strong explanatory factor for VSC generation. These observations support analyzing low-flow subgroups and oral inflammatory markers when evaluating treatment response [4].

A persistent challenge in halitosis research is endpoint heterogeneity. Organoleptic assessment remains clinically intuitive and captures human-perceived malodor, but it is inherently subjective and judge-dependent; nonetheless, standardized 0–5 organoleptic scales have been validated against odorant concentrations. Portable sulfide monitors provide rapid, chairside biochemical proxies and show meaningful correlation with organoleptic ratings, supporting their usefulness for pragmatic trials. Gas chromatography, including portable systems designed for breath malodor assessment, allows compound-specific profiling and is often treated as a reference method for mechanistic inference [5,6,7].

Mechanism-based management strategies align with these etiologic domains. Mechanical tongue cleaning directly targets the dominant niche for odorant production; in a crossover clinical trial, a tongue scraper achieved a larger reduction in VSCs than a toothbrush (75% vs. 45%), and systematic review evidence supports tongue cleaning as an effective component of halitosis care. Beyond odor intensity, halitosis-specific quality-of-life instruments capture patient-relevant impact that odor measures alone may miss. Additionally, reflux symptoms can co-occur with self-reported halitosis in population studies, supporting reflux screening as a contextual variable in clinic-based trials. Finally, zinc-based rinses act via sulfur binding and VSC neutralization, and zinc–chlorhexidine formulations have demonstrated superior anti-halitosis efficacy compared with some existing formulations, while controlled trials of multiple rinse products (including zinc-containing regimens) show measurable short-term improvements in intra-oral halitosis [8,9,10,11,12,13].

Probiotics represent a complementary ecological strategy: rather than broadly suppressing oral bacteria, they aim to promote a less odorogenic tongue biofilm. Among candidate strains, Streptococcus salivarius K12 (BLIS K12^®^) has randomized placebo-controlled evidence for improving tongue coating-associated halitosis and it is delivered as a slowly dissolving lozenge/tablet to maximize oral contact time [14]. Zinc-based mouthrinses reduce malodor through VSC binding/neutralization and, depending on the formulation, may also reduce odorant production by lowering anaerobic bacterial load. In routine care, one widely used anti-halitosis formulation combines zinc lactate with low-dose chlorhexidine and cetylpyridinium chloride, balancing efficacy with improved tolerability compared with higher-dose chlorhexidine regimens [12,13].

Against this background, we designed a study comparing (i) standardized counseling alone, (ii) daily S. salivarius K12 probiotic lozenges, and (iii) a zinc lactate/CPC/low-dose chlorhexidine mouthrinse over 4 weeks, using endpoints feasible in standard dental practice (portable sulfide monitoring, organoleptic scoring, and validated patient-reported instruments), with microbiological enrichment via salivary *So. moorei* quantification.

## 2. Materials and Methods

### 2.1. Study Design and Settings

This was a 4-week, parallel-group, prospective study conducted in a university-affiliated dental clinic within the “Victor Babeș” University of Medicine and Pharmacy Timișoara. The design prioritized pragmatic feasibility: short follow-up, simple interventions, and endpoints that can be collected during routine preventive visits. The study was structured to resemble “real clinic” constraints while still applying core elements of rigorous comparative research (random allocation, standardized measurement, and prespecified analyses). This investigator-initiated trial was not prospectively registered before the first enrollment. We acknowledge this as a methodological limitation. A written protocol and a prespecified statistical analysis plan had been prepared before database closing and they are available from the corresponding authors upon reasonable request.

Approval for the research was obtained from the Institutional Research Board, adhering to the principles outlined in the Declaration of Helsinki (approval code E-787, dated 8 February 2023). Participant confidentiality was maintained via coded identifiers and restricted access to the study dataset. Staff members who performed organoleptic scoring and VSC readings were blinded to treatment allocation; the participants were instructed not to disclose their group to the examiners during follow-up visits.

This investigator-initiated trial was not prospectively registered. A finalized protocol and prespecified statistical analysis plan (SAP) were locked prior to database closing and they are available from the corresponding authors upon reasonable request. The SAP prespecified the primary endpoint (change in total VSCs from baseline to week 4), the main comparative model (ANCOVA adjusted for baseline values), and the interpretation of the logistic regression as exploratory rather than confirmatory. No changes were made to the primary endpoint, primary timepoint, or the three-arm randomization design after study initiation.

**Figure 1:**
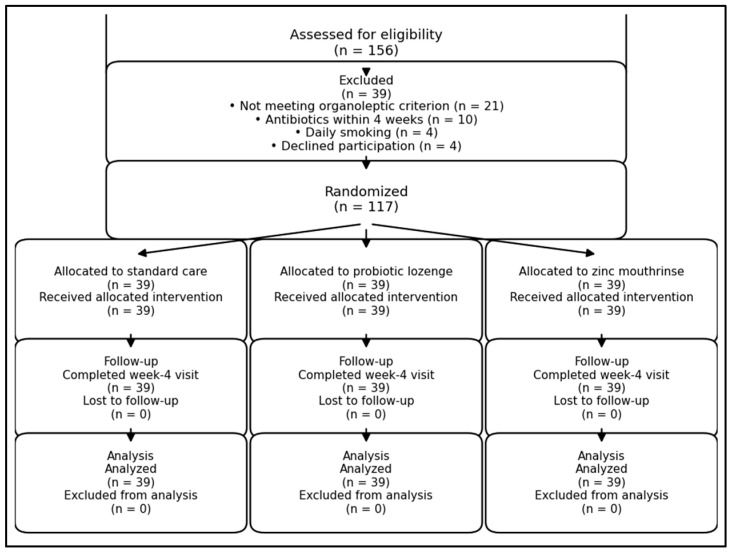
CONSORT participant flow diagram.

The sample size was planned for the primary outcome (change in VSC, ppb) across three groups. Based on prior chairside VSC studies and clinic feasibility, we targeted the detection of a 40 ppb between-group difference in mean VSC change (zinc vs. standard care) with an assumed SD of 60 ppb, corresponding to a standardized effect size of 0.67. With α = 0.05 and 90% power for a three-arm comparison, 36 participants per group were required; allowing for 8% attrition/non-evaluable follow-up, we enrolled 39 individuals per group (n = 117), as presented in Figure 1.

The participants could not be blinded due to distinct interventions and absence of placebo products. Accordingly, the study should be interpreted as an open-label trial with blinded outcome assessment rather than a fully blinded randomized trial. To mitigate expectancy/performance bias, (i) assessors were blinded, (ii) participants were instructed not to disclose allocation, (iii) counseling was delivered via a fixed script, and (iv) objective (VSCs) and assessor-rated (organoleptic) outcomes were prioritized in inference, with patient-reported outcomes interpreted as supportive. Accordingly, inference was anchored on objective (VSCs) and blinded assessor-rated (organoleptic) outcomes, while patient-reported endpoints (HALT, OHIP-14) were interpreted as supportive and potentially susceptible to expectancy effects.

### 2.2. Participants and Eligibility

We enrolled 117 participants presenting for preventive care who reported bothersome halitosis for at least 3 months and met a screening criterion of baseline organoleptic score ≥ 2. Eligible ages were 18–40 years. Recruitment was consecutive, and eligibility was confirmed in a standardized intake process before randomization. Exclusion criteria were selected to reduce major non-oral contributors and recent exposures likely to distort malodor metrics: acute upper respiratory infection within 10 days; antibiotics within 4 weeks; daily smoking; uncontrolled systemic illness associated with breath changes (uncontrolled diabetes, advanced hepatic/renal failure); current chlorhexidine use; and pregnancy. The participants were asked to avoid pungent foods and alcohol for 12 h and to avoid oral hygiene procedures, chewing gum, and breath mints for 2 h before each assessment. These restrictions were designed to stabilize short-term odor fluctuations and improve comparability across visits.

### 2.3. Intervention Arms and Study Procedures

Participants were assigned 1:1:1 to: (A) standard care, (B) probiotic lozenge, or (C) zinc mouthrinse (39 participants per arm), in a randomized fashion. Randomization used a computer-generated schedule with randomly permuted block sizes of 3 and 6 to maintain allocation balance while limiting predictability. No stratification was used. Allocation concealment was implemented using sequentially numbered, opaque, sealed envelopes prepared by a staff member not involved in recruitment or assessment. After eligibility confirmation and baseline measurements, the coordinator opened the next envelope and dispensed the assigned intervention.

All groups received standardized counseling (10 min) delivered by trained staff using a scripted checklist: twice daily toothbrushing technique, interdental cleaning options, diet triggers (pungent foods/alcohol), hydration guidance, and tongue dorsum cleaning instructions (demonstration of gentle posterior-to-anterior scraping for 10–15 s once daily). The participants were asked not to start new oral care products during follow-up and to maintain their baseline brushing/flossing frequency. Standard care (active behavioral control): standardized counseling and tongue cleaning instruction only, without additional antimicrobial/probiotic products. Probiotic arm: BLIS Technologies Ltd. (Dunedin, New Zealand) DailyDefence^®^ lozenges containing Streptococcus salivarius K12 (BLIS K12^®^), ≥1.25 × 10^9^ CFU per lozenge at manufacture. The participants dissolved one lozenge nightly after brushing and avoided food/drink for 20 min afterward. Zinc mouthrinse arm: Halita^®^ mouthwash (DENTAID, Barcelona, Spain) containing chlorhexidine digluconate 0.05%, cetylpyridinium chloride 0.05%, and zinc lactate 0.14% (alcohol-free). The participants rinsed with 15 mL for 30 s twice daily (morning/evening) after brushing, then spat without water rinsing and avoided food/drink for 30 min.

Responder thresholds were prespecified as pragmatic, distribution-based cutoffs chosen to reflect clinically noticeable short-term improvement: Δorganoleptic ≤ −1.0, ΔHALT ≤ −10, and ΔVSC ≤ −50 ppb. Responder analyses are presented as exploratory summaries complementing continuous outcome comparisons.

The Rationale for Primary Endpoint Selection: We selected change in total VSCs measured by portable sulfide monitoring as the primary comparative endpoint to align the trial with routine dental practice, where chairside, time-efficient, standardized measurements are feasible at scale. Portable sulfide monitoring provides an objective, repeatable proxy for intra-oral malodor that correlates with organoleptic assessment and that has been widely used in clinical halitosis research. We acknowledge that gas chromatography provides compound-specific information and stronger mechanistic inference; therefore, the present study emphasizes comparative short-term effectiveness in a pragmatic setting rather than detailed odorant profiling. We intentionally used an active behavioral control to reflect ethically appropriate usual care and to estimate the incremental benefit of product-based strategies beyond standardized counseling. For clarity, the manuscript now uses “VSCs” when referring to measured volatile sulfur compounds or their levels and “VSC” only when used adjectivally (Table 1).

VSC (Portable Sulfide Monitor): VSCs were measured using Halimeter^®^ RH17K (Interscan Corporation, Chatsworth, CA, USA). The device was warmed up per manufacturer instructions and calibrated daily using a certified hydrogen sulfide standard. The measurements were performed between 09:00 and 11:00, in a ventilated room free of strong odors. The participants kept the mouth closed for 60 s and then exhaled gently through a disposable straw positioned at the device inlet; three readings separated by 60 s were recorded and averaged for analysis.

Organoleptic Scoring: Two calibrated dentists independently rated breath odor on a 0–5 scale in 0.5-point increments (0 none; 5 severe) at a fixed distance after 60 s of mouth closure. The mean of two raters was used. Before the enrollment, the raters completed standardized training and achieved weighted κ = 0.78 and ICC(2,1) = 0.84 on 20 pilot assessments.

Unstimulated saliva (2 mL) was collected into sterile tubes, placed on ice, and stored at −80 °C within 2 h. DNA was extracted using a silica-membrane kit (QIAamp DNA Mini Kit, QIAGEN, Hilden, Germany). Quantitative PCR targeted *So. moorei* 16S rRNA using the following primers: forward 5′-CTCAACCGGGGAGGGT-3′ and reverse 5′-CGGGTATCTAACGCAGTACTC-3′, with SYBR-green detection. Standard curves were generated from serial dilutions (10^2^–10^8^ copies/reaction), and the results were expressed as log10 copies/mL. Negative controls (no-template) and inhibition checks were included in each run (Table 2).

### 2.4. Statistical Analysis

The statistical analysis used two-sided α = 0.05. Baseline comparability was assessed with one-way ANOVA (approximately normal continuous variables), Kruskal–Wallis tests (skewed variables such as VSCs), and χ^2^ tests for categorical variables. The primary inferential framework compared between-arm change using ANCOVA (change ~ arm + baseline value), improving precision and handling small baseline differences. VSCs were log-transformed for ANCOVA due to right-skew. Spearman correlations summarized cross-domain associations at baseline. A multivariable logistic regression modeled the odds of moderate/severe halitosis at week 4 (organoleptic ≥ 2.5) using arm assignment and baseline predictors. The primary analysis followed an intention-to-treat principle including all randomized participants by assigned arm. There were no missing observations for the primary outcome at week 4; secondary outcomes were also complete. Because the study was powered for the primary endpoint, all secondary endpoint analyses, responder summaries, subgroup comparisons, and the logistic regression were interpreted as supportive or exploratory and were not multiplicity-adjusted. To assess the stability of the exploratory logistic model and reduce optimism in performance estimates, we additionally performed internal validation using bootstrap resampling (1000 resamples) to obtain an optimism-corrected AUC and calibration slope. These analyses were considered sensitivity checks and were not used for confirmatory inference. In bootstrap internal validation, model discrimination remained in a similar range, but calibration showed expected optimism, supporting the interpretation of the model as exploratory and hypothesis-generating.

## 3. Results

Across the three randomized arms, the participants were well balanced at baseline with no statistically significant differences in demographics, oral hygiene behaviors, or key contextual factors (all *p* ≥ 0.27). Mean age was ~25–26 years (25.7 ± 4.6 standard care; 25.8 ± 4.3 probiotic; 25.3 ± 4.9 zinc; *p* = 0.885), and ~59–64% of participants were female (*p* = 0.88), with comparable BMI (23.6–24.1 kg/m^2^; *p* = 0.818). Self-reported behaviors were similar, including brushing ≥ 2/day (64.2–66.7%; *p* = 0.964), daily flossing (38.6–43.7%; *p* = 0.883), and daily tongue cleaning (33.4–38.6%; *p* = 0.882). Frequent snacking was reported by ~44–54% (*p* = 0.638). The reflux symptom scores were modest and not different between groups (4.0 ± 2.4 vs. 4.8 ± 2.1 vs. 4.7 ± 2.3; *p* = 0.27), and the prevalence of low unstimulated salivary flow was ~26–28% in all arms (28.3% vs. 25.8% vs. 25.8%; *p* = 0.946), supporting good baseline comparability prior to intervention (Table 3). Because participants were not placebo-blinded, patient-reported outcomes are presented as clinically relevant supportive endpoints but may be more susceptible to expectancy-related bias than objective VSC and blinded organoleptic assessments.

Baseline oral clinical indices, objective malodor measures, patient-reported outcomes, and the microbiological marker were closely matched among groups, with no significant between-arm differences (all *p* ≥ 0.697). Plaque index averaged 1.6–1.7 and gingival index averaged 1.4 across arms (both *p* ≈ 0.915–0.916); the tongue coating score was identical at 1.7 ± 0.7 (*p* = 0.962). Unstimulated salivary flow was similar (0.3 ± 0.2 mL/min in all groups; *p* = 0.886). The VSC levels were comparable by median [IQR] (178.7 [138.2–236.6] standard care; 171.2 [132.6–238.8] probiotic; 168.2 [137.7–220.5] zinc; *p* = 0.697), as were the organoleptic scores (3.1 [2.6–3.6], 3.1 [2.6–3.5], 3.0 [2.5–3.6]; *p* = 0.842). Halitosis-specific burden and oral health-related quality of life were also similar (HALT ~59–60; OHIP-14 ~20.4–20.6; *p* ≥ 0.835), and *So. moorei* abundance was identical (6.4 ± 0.6 log10 copies/mL; *p* = 0.87), indicating equivalent baseline disease severity and mechanistic correlates (Table 4).

All groups improved over 4 weeks, but the magnitude of improvement followed a clear gradient (zinc > probiotic > standard care) with significant between-arm differences for every endpoint reported (*p* ≤ 0.033), including the clinical binary outcome. Objective malodor improved modestly with standard care (ΔVSC −12.7 ± 33.9 ppb) but substantially with probiotics (Δ −47.3 ± 42.2 ppb) and the most with zinc (Δ −78.5 ± 36.3 ppb; *p* < 0.001). Clinician-rated malodor decreased by Δ −0.2 ± 0.7 (standard), Δ −0.8 ± 0.8 (probiotic), and Δ −1.2 ± 0.8 (zinc; *p* < 0.001). Patient-reported impact improved similarly (HALT: Δ −9.9 ± 7.8 vs. −15.7 ± 8.0 vs. −20.9 ± 8.0; *p* < 0.001; OHIP-14: Δ −2.9 ± 3.2 vs. −5.0 ± 3.3 vs. −6.8 ± 3.7; *p* < 0.001). Mechanistic correlates shifted in parallel: tongue coating decreased (Δ −0.3 ± 0.4 vs. −0.5 ± 0.4 vs. −0.7 ± 0.4; *p* < 0.001), plaque decreased slightly (Δ −0.1 ± 0.3 vs. −0.2 ± 0.3 vs. −0.3 ± 0.3; *p* = 0.033), and *So. moorei* declined most in probiotic and zinc arms (Δ −0.6 ± 0.5 and −0.7 ± 0.5 log10 copies/mL vs. −0.2 ± 0.5; *p* < 0.001). Importantly, moderate/severe halitosis at week 4 (organoleptic ≥ 2.5) remained common with standard care (58.9%) and probiotics (46.2%) but was markedly lower with zinc (20.5%; *p* = 0.004), as seen in Table 5.

Baseline correlations supported coherence across objective odor, clinician ratings, patient impact, and oral ecological drivers. VSC showed a strong positive association with organoleptic scoring (ρ = 0.7, *p* < 0.001) and moderate correlations with tongue coating (ρ = 0.5, *p* < 0.001), HALT (ρ = 0.4, *p* < 0.001), OHIP-14 (ρ = 0.3, *p* < 0.001), plaque (ρ = 0.3, *p* < 0.001), and *So. moorei* (ρ = 0.3, *p* < 0.001), while correlating inversely with salivary flow (ρ = −0.4, *p* < 0.001). Organoleptic scores mirrored this pattern, correlating with HALT (ρ = 0.5, *p* < 0.001), OHIP-14 (ρ = 0.4, *p* < 0.001), tongue coating (ρ = 0.4, *p* < 0.001), and inversely with salivary flow (ρ = −0.4, *p* < 0.001). Patient-reported burden (HALT) correlated strongly with OHIP-14 (ρ = 0.5, *p* < 0.001) and showed modest relationships with reflux symptoms (ρ = 0.3, *p* < 0.001), as seen in Table 6.

An exploratory multivariable logistic regression evaluated predictors of residual moderate/severe halitosis at week 4 (organoleptic ≥ 2.5). To reduce overfitting risk and to improve interpretability, predictors were prespecified and primarily dichotomized: treatment arm, low salivary flow (<0.25 mL/min), high reflux symptoms (score ≥ 6), baseline organoleptic > 3.0, and tongue coating ≥ 2. No stepwise selection was used (Table 7).

When stratified by salivary flow, treatment effects remained directionally consistent, and formal interaction testing did not support effect modification by flow status. In the low-flow subgroup, zinc produced the largest reductions in VSC (Δ −57.4 ± 32.8 ppb) compared with probiotics (Δ −25.2 ± 37.5) and standard care (Δ +6.3 ± 33.7), with a significant between-arm difference (*p* = 0.041). Organoleptic improvement also favored zinc (Δ −0.9 ± 0.7) and probiotics (Δ −0.6 ± 0.7) versus standard care (Δ −0.1 ± 0.7; *p* = 0.017), while HALT changes were numerically largest with zinc (Δ −15.2 ± 9.6) but did not reach significance (*p* = 0.075). In the normal-flow subgroup, between-arm differences were pronounced and highly significant across all three outcomes: VSC (Δ −20.2 ± 33.2 standard vs. −55.1 ± 42.7 probiotic vs. −85.7 ± 34.0 zinc; *p* < 0.001), organoleptic (Δ −0.2 ± 0.7 vs. −0.9 ± 0.8 vs. −1.3 ± 0.8; *p* < 0.001), and HALT (Δ −11.4 ± 7.0 vs. −17.5 ± 7.0 vs. −22.8 ± 7.0; *p* < 0.001). Interaction *p* values (0.428 for VSC; 0.946 for organoleptic; 0.654 for HALT) indicated no statistically significant arm × flow interaction, suggesting broadly similar relative benefits across the salivary flow strata (Table 8).

**Figure 2:**
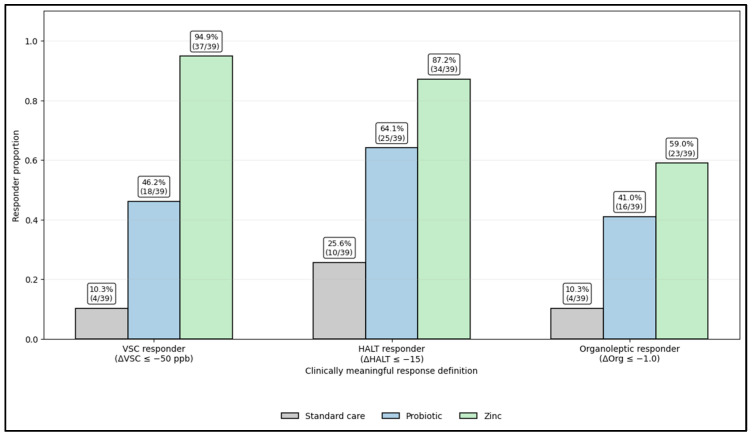
Clinically meaningful response rates at 4 weeks by randomized intervention arm (ΔVSC, ΔHALT, and Δorganoleptic thresholds).

Figure 2 summarizes responder proportions using three clinically intuitive thresholds, showing a clear dose–response pattern across arms. Zinc produced the highest response rates for all endpoints: VSC responders 94.9% (37/39), HALT responders 87.2% (34/39), and organoleptic responders 59.0% (23/39). Probiotic showed intermediate benefit—46.2% (18/39) VSC responders, 64.1% (25/39) HALT responders, and 41.0% (16/39) organoleptic responders—while standard care had the lowest response rates (VSC 10.3% (4/39); HALT 25.6% (10/39); organoleptic 10.3% (4/39)). Separation was the most pronounced for objective odor reduction (ΔVSC) and remained consistent across patient-reported burden (ΔHALT) and clinician scoring (Δorganoleptic), supporting strong between-arm differences in clinically meaningful improvement.

**Figure 3:**
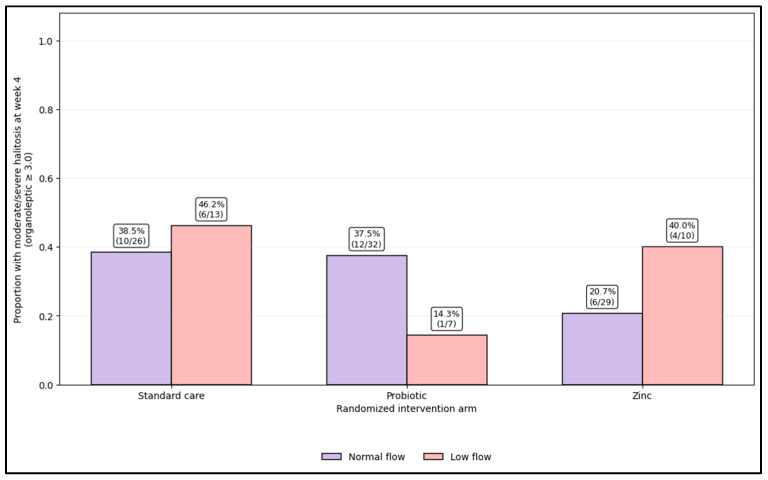
Week 4 moderate/severe halitosis prevalence (organoleptic ≥ 2.5) by intervention arm, stratified by salivary flow status.

Figure 3 displays week 4 moderate/severe halitosis (organoleptic ≥ 2.5) by arm with stratification by salivary flow, highlighting subgroup patterns that are clinically interpretable. In the standard care group, prevalence was similar in the normal-flow and low-flow participants (38.5% (10/26) vs. 46.2% (6/13)). In the probiotic group, prevalence was notably lower among the low-flow participants (14.3% (1/7)) compared with the normal-flow participants (37.5% (12/32)). In the zinc group, the normal-flow participants had the lowest prevalence overall (20.7% (6/29)), but the low-flow participants showed higher residual risk (40.0% (4/10)). Overall, the plot emphasizes that low salivary flow can coincide with higher week 4 malodor burden in some arms (notably zinc), while zinc still yields the lowest observed prevalence among the normal-flow participants. Residual moderate/severe halitosis was defined as week 4 organoleptic ≥ 2.5, reflecting a clinically interpretable threshold when using the average of two blinded 0.5-increment ratings.

**Figure 4:**
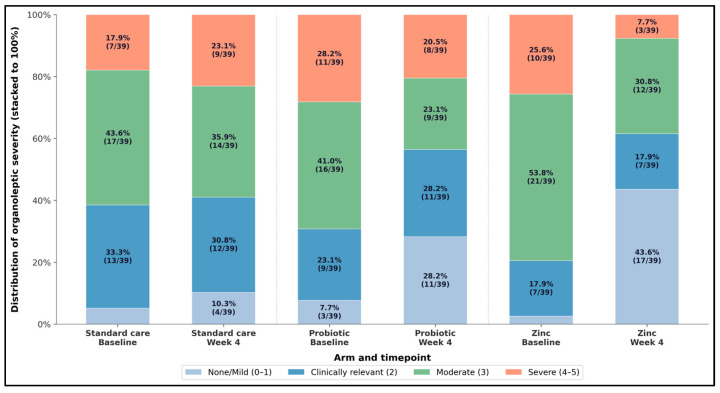
Shift in organoleptic severity distribution from baseline to week 4 across intervention arms (stacked 100% bars).

Figure 4 visualizes the full distributional shift in organoleptic severity from baseline to week 4, providing a more granular picture than a single cutoff. Zinc showed the most favorable redistribution: the none/mild category increased from 2.6% (1/39) at baseline to 43.6% (17/39) at week 4, while the severe category decreased from 25.6% (10/39) to 7.7% (3/39). The probiotic group also improved the distribution, with none/mild rising from 7.7% (3/39) to 28.2% (11/39) and moderate decreasing from 41.0% (16/39) to 23.1% (9/39). Standard care showed smaller net improvement and a modest increase in severe category (17.9% (7/39) to 23.1% (9/39)). This figure supports the view that intervention effects are not limited to crossing a single threshold but reflect broader shifts toward lower clinician-rated malodor severity, the strongest for zinc.

Table 9 shows that both active interventions produced greater improvements than standard care across all outcomes (negative Δdiff values), with zinc consistently achieving the largest benefits. Compared with the standard, zinc reduced VSC by −65.8 ppb and improved organoleptic scores (−1.0), HALT (−11.0), and OHIP-14 (−3.9), while probiotic also improved these measures but to a lesser extent (e.g., VSC −34.6 ppb; organoleptic −0.6; HALT −5.8; OHIP-14 −2.1). Directly comparing zinc vs. probiotic, zinc provided additional meaningful reductions in VSC (−31.2), organoleptic score (−0.4), HALT (−5.2), and OHIP-14 (−1.8), whereas between-group differences were smaller and borderline for tongue coating, plaque index, and *So. moorei* (with confidence intervals approaching or crossing 0 for some comparisons).

## 4. Discussion

In this study, we observed a consistent pattern of larger mean improvements in the zinc mouthrinse arm compared with the probiotic arm, with both arms improving more than standardized counseling alone. The zinc arm achieved the greatest mean VSC reduction (Δ −78.5 ppb) and the largest organoleptic improvement (Δ −1.2 on a 0–5 scale), accompanied by the greatest decreases in HALT (Δ −20.9) and OHIP-14 (Δ −6.8). This pattern is consistent with clinical studies showing that zinc-containing regimens can meaningfully reduce oral malodor, including zinc lactate toothpaste/rinse protocols and adjunct approaches that combine chemical control with mechanical tongue measures [15,16,17].

Mechanistically, the concordant improvements across VSC, organoleptic scores, tongue coating, and *So. moorei* burden support a tongue biofilm-centered interpretation in this cohort. At baseline, tongue coating correlated more strongly with odor outcomes than plaque, and both active interventions reduced tongue coating (zinc Δ −0.7; probiotic Δ −0.5) alongside larger drops in salivary *So. moorei* (zinc Δ −0.7 log10; probiotic Δ −0.6 log10). This aligns with microbiological work implicating Solobacterium moorei and tongue dorsum community structure in clinically relevant halitosis phenotypes, while also reinforcing that halitosis is polymicrobial and best explained by ecological niches rather than a single organism [18,19]. At the same time, salivary qPCR for *So. moorei* provides only a narrow and indirect window into tongue ecology, so the present microbiological findings should not be interpreted as a comprehensive description of the odorogenic microbiome. Future studies should therefore incorporate broader tongue dorsum microbiome profiling, ideally with longitudinal sampling before, during, and after treatment, to determine whether short-term odor reduction corresponds to sustained ecological remodeling. Because the control arm included evidence-based behavioral counseling and tongue hygiene instruction, between-arm differences should be interpreted as incremental effects beyond standardized care; this design likely attenuated effect sizes relative to a no-treatment control.

The subgroup and multivariable analyses also highlight why “the same product” can perform differently across patients: low unstimulated salivary flow was a strong predictor of residual moderate/severe halitosis at week 4 (OR 2.9), and low-flow participants had attenuated responses—especially with counseling alone, where mean VSC change was minimal/worse (+6.3 ppb). This is consistent with evidence that resting salivary flow is a meaningful modulating factor for oral malodor, plausibly via reduced clearance of odorants and slower disruption of anaerobic niches between hygiene events. Clinically, these findings support bundling (i) a high-impact anti-odor strategy (zinc performed best here) with (ii) saliva-supportive measures and review of xerostomia drivers when baseline flow is low [20].

The probiotic arm’s intermediate effect (VSC Δ −47.3 ppb; organoleptic Δ −0.8) fits the broader literature that probiotics can improve halitosis outcomes in some settings, but with strain-, delivery-, and context-dependence. A randomized placebo-controlled trial of Weissella cibaria CMU tablets reported reductions in halitosis measures over a similar short timeframe, and a randomized trial in severe periodontitis showed that adjunct Lactobacillus therapy improved halitosis alongside periodontal management. Meanwhile, a time-perspective meta-analysis suggests probiotic benefit is most evident in the short term (≤4 weeks), which matches the clinic-friendly window used in this study and supports the interpretation of probiotics as an ecological modulator rather than an immediate chemical neutralizer [21,22,23]. However, precisely because probiotic strategies aim to reshape the oral ecosystem rather than simply neutralize odorants acutely, the present 4-week design cannot determine whether benefits would persist after the discontinuation, plateau with longer use, or relapse once the product use is stopped. This issue is equally relevant for zinc-based regimens, which may provide strong short-term odor suppression during active use without necessarily producing durable microbiome shifts. Longer post-treatment follow-up is therefore essential to distinguish transient control from sustained remission.

Finally, the reflux signal in our model (high reflux symptoms OR 4.0 for residual moderate/severe halitosis) is best read as a context marker rather than a definitive extra-oral cause in most young adults with intra-oral disease signatures. A systematic review of epidemiological surveys concluded that halitosis can appear among extra-esophageal manifestations reported in GERD populations, but objective associations are inconsistent; for example, a study using both questionnaire-based and Halimeter-based halitosis measures found that GERD might not correlate with objective halitosis despite relationships between “informed” (perceived) and measured odor. A practical implication is to screen reflux symptoms to identify patients who may need parallel management (dietary triggers, nocturnal reflux control, and mouth-breathing/sleep factors) to reduce persistent complaints even when intra-oral metrics improve [24,25,26,27].

Clinically, these findings support that a zinc-containing anti-halitosis mouthrinse can be a useful short-term adjunct to standardized counseling for patients with persistent intra-oral halitosis, while S. salivarius K12 probiotic lozenges may offer a smaller, intermediate benefit. Because participants were not placebo-blinded and follow-up was limited to 4 weeks, patient-reported improvements should be interpreted cautiously and durability beyond the study window remains unknown. The reflux symptom association observed in exploratory models should be viewed as a context marker rather than evidence of GERD causality, as symptoms were not clinically verified. In practice, these findings most directly inform short-term adjunct management of persistent intra-oral halitosis in young adult preventive care patients within similar outpatient settings, rather than broader populations with higher comorbidity, advanced periodontitis, or severe xerostomia. The study also adds region-specific evidence from a Romanian university-affiliated outpatient setting, where diet, oral hygiene routines, health-seeking behavior, and prior familiarity with probiotic or antiseptic products may differ from populations examined in earlier trials. For that reason, extrapolation beyond similar young adult outpatient populations should still be made with caution. Nevertheless, these findings should be interpreted in light of potential residual confounding from unmeasured or incompletely controlled factors, including underlying comorbidities and other patient- and treatment-related characteristics [28,29,30,31,32,33,34,35].

Regarding limitations, the study was not prospectively registered. Although a protocol and SAP were finalized prior to database closing, a lack of prospective registration increases the risk of perceived selective outcome reporting and limits the strength of causal claims that can be made from a pragmatic trial. This study was conducted at a single university clinic with a relatively young adult sample (18–40 years), limiting generalizability to older populations with higher periodontal and xerostomia burden. The follow-up was short (4 weeks), so the durability of benefit and relapse patterns are unknown. While the assessors were blinded, participant blinding and placebo control were not implemented, leaving room for expectation effects, particularly for patient-reported outcomes (HALT, OHIP-14). VSCs were measured via portable halitometry to maximize pragmatic feasibility; however, compared with gas chromatography, this approach does not provide compound-specific profiling and limits mechanistic inference regarding individual odorants. Adherence was monitored pragmatically (diaries, blister counts, bottle weight differences) but remains imperfect. The absence of placebo products and participant blinding may have amplified perceived improvements (HALT, OHIP-14) and could also have influenced behavior-dependent measures despite standardized instructions. These considerations are less likely to fully explain the consistent gradient observed in objective VSC and blinded organoleptic outcomes, but they reduce certainty regarding the magnitude of benefit for patient-reported and behavior-sensitive endpoints. Finally, microbiological assessment focused on salivary *So. moorei* (qPCR) and did not characterize the broader tongue microbiome or other odorogenic taxa. Because saliva is only a partial proxy for tongue dorsum ecology, this limits mechanistic interpretation of how each intervention altered the polymicrobial communities most directly involved in malodor generation. The short intervention window also prevents any inference about the persistence of benefits after treatment discontinuation or the timing and magnitude of relapse. Future registered trials should incorporate post-treatment follow-up and broader tongue dorsum microbiome profiling to determine whether short-term odor reduction translates into sustained ecological change.

## 5. Conclusions

In this 4-week randomized, assessor-blinded pragmatic trial in young adults, both probiotic lozenges and a zinc-containing mouthrinse were associated with improvements in intra-oral halitosis metrics beyond standardized counseling alone. The zinc formulation showed larger short-term improvements in objective VSC reduction and assessor-rated organoleptic scores than the probiotic lozenge within this trial context. Because participants were not placebo-blinded and follow-up was limited to 4 weeks, the magnitude of patient-reported benefits may be influenced by expectancy effects and durability beyond the study window remains uncertain. These findings support cautious short-term adjunct use in similar outpatient dental populations and motivate longer, placebo-controlled trials assessing durability and patient-centered outcomes. Future prospectively registered, placebo-controlled studies with longer post-discontinuation follow-up and broader tongue microbiome characterization are needed to define sustainability, relapse patterns, regional generalizability, and mechanisms.

## Figures and Tables

**Table 1:** Intervention specifications and adherence monitoring.

Arm	Product (Manufacturer)	Active Components (Per Label)	Dosing Regimen	Key Use Instructions	Adherence Measure
Standard care	Counseling script (study staff)	No reported	Single standardized session at baseline	Toothbrushing + interdental cleaning + tongue cleaning demonstration; no new products	Week 4 checklist of adherence to counseling behaviors
Probiotic lozenge	DailyDefence^®^ BLIS K12^®^ (BLIS Technologies Ltd., Dunedin, New Zealand)	*S. salivarius* K12 ≥ 1.25 × 10^9^ CFU/lozenge	1 lozenge nightly × 4 weeks	Dissolve slowly after brushing; no food/drink × 20 min	Blister count + daily calendar
Zinc mouthrinse	Halita^®^ (DENTAID, Barcelona, Spain)	CHX 0.05% + CPC 0.05% + zinc lactate 0.14%	15 mL × 30 s twice daily × 4 weeks	Spit; no water rinse; no food/drink × 30 min	Bottle weight change + daily diary

**Table 2:** Measurement protocols and instruments.

Domain	Instrument/Scale	Protocol Summary
VSC	Halimeter^®^ RH17K	3 readings/timepoint; averaged; morning window; daily calibration
Organoleptic	0–5, 0.5 increments	Two blinded raters; mean score used; fixed distance after 60 s mouth closure
Tongue coating	0–3 clinical score	Visual tongue dorsum score under standard light
Plaque	0–3 plaque index	Standard plaque scoring across index teeth
Gingival inflammation	0–3 gingival index	Standard gingival index scoring
Salivary flow	Passive drool (5 min)	Unstimulated whole saliva; mL/min; low flow < 0.25 mL/min
Patient impact	HALT; OHIP-14	Self-administered validated questionnaires
Microbiology	qPCR for *So. moorei*	Saliva collection + DNA extraction + 16S qPCR; log10 copies/mL
Reflux symptoms	0–12 score	3 items (heartburn, regurgitation, and throat irritation), each 0–4

**Table 3:** Baseline participant characteristics and behaviors.

Characteristic	Standard Care (n = 39)	Probiotic Lozenge (n = 39)	Zinc Mouthrinse (n = 39)	*p* Value
Age, years	25.7 ± 4.6	25.8 ± 4.3	25.3 ± 4.9	0.885
Female sex	24 (61.6)	25 (64.2)	23 (58.9)	0.88
BMI, kg/m^2^	23.6 ± 3.3	24.1 ± 3.5	23.8 ± 3.2	0.818
Brushing ≥ 2/day	25 (64.2)	26 (66.7)	26 (66.7)	0.964
Daily flossing	15 (38.6)	17 (43.7)	17 (43.7)	0.883
Daily tongue cleaning (baseline)	14 (35.9)	15 (38.6)	13 (33.4)	0.882
Frequent snacking (≥3/day)	19 (48.8)	21 (54.0)	17 (43.7)	0.638
Reflux symptom score (0–12)	4.0 ± 2.4	4.8 ± 2.1	4.7 ± 2.3	0.27
Low salivary flow (<0.25 mL/min)	11 (28.3)	10 (25.8)	10 (25.8)	0.946

Data are shown as mean ± SD or n (%). BMI, body mass index; mL/min, milliliters per minute.

**Table 4:** Baseline clinical, biochemical, and microbiological measures.

Clinical Measure	Standard Care (n = 39)	Probiotic Lozenge (n = 39)	Zinc Mouthrinse (n = 39)	*p* Value
Plaque index (0–3)	1.6 ± 0.5	1.6 ± 0.6	1.7 ± 0.6	0.915
Gingival index (0–3)	1.4 ± 0.5	1.4 ± 0.4	1.4 ± 0.5	0.916
Tongue coating score (0–3)	1.7 ± 0.7	1.7 ± 0.7	1.7 ± 0.7	0.962
Unstimulated salivary flow, mL/min	0.3 ± 0.2	0.3 ± 0.2	0.3 ± 0.2	0.886
VSC level (ppb)	178.7 [138.2–236.6]	171.2 [132.6–238.8]	168.2 [137.7–220.5]	0.697
Organoleptic score (0–5)	3.1 [2.6–3.6]	3.1 [2.6–3.5]	3.0 [2.5–3.6]	0.842
HALT total score (0–100)	60.0 ± 9.0	58.8 ± 9.7	59.9 ± 9.9	0.835
OHIP-14 total score (0–56)	20.6 ± 5.5	20.6 ± 5.9	20.4 ± 5.1	0.986
*So. moorei* (log10 copies/mL)	6.4 ± 0.6	6.4 ± 0.6	6.4 ± 0.6	0.87

Data are shown as mean ± SD or median [IQR]. VSCs, volatile sulfur compounds; ppb, parts per billion; HALT, Halitosis Associated Life-Quality Test (0–100); OHIP-14, Oral Health Impact Profile-14 (0–56); *So. moorei*, *Solobacterium moorei*; mL/min, milliliters per minute; IQR, interquartile range.

**Table 5:** Outcomes at baseline and week 4 and change from baseline.

Outcome	Standard Care	Probiotic Lozenge	Zinc Mouthrinse	*p* (Δ Between Arms)
VSC level (ppb)	Baseline 185.6 ± 64.2	Baseline 179.3 ± 65.2	Baseline 179.8 ± 68.3	<0.001
	Week 4 172.9 ± 70.2	Week 4 132.0 ± 52.6	Week 4 101.3 ± 41.6	
	Δ −12.7 ± 33.9	Δ −47.3 ± 42.2	Δ −78.5 ± 36.3	
Organoleptic score (0–5)	Baseline 3.0 ± 0.8	Baseline 3.0 ± 0.8	Baseline 3.0 ± 0.9	<0.001
	Week 4 2.8 ± 0.9	Week 4 2.2 ± 0.9	Week 4 1.8 ± 0.8	
	Δ −0.2 ± 0.7	Δ −0.8 ± 0.8	Δ −1.2 ± 0.8	
HALT total score (0–100)	Baseline 60.0 ± 9.0	Baseline 58.8 ± 9.7	Baseline 59.9 ± 9.9	<0.001
	Week 4 50.0 ± 11.1	Week 4 43.1 ± 11.6	Week 4 39.0 ± 11.4	
	Δ −9.9 ± 7.8	Δ −15.7 ± 8.0	Δ −20.9 ± 8.0	
OHIP-14 total score (0–56)	Baseline 20.6 ± 5.5	Baseline 20.6 ± 5.9	Baseline 20.4 ± 5.1	<0.001
	Week 4 17.7 ± 6.4	Week 4 15.6 ± 6.1	Week 4 13.6 ± 5.9	
	Δ −2.9 ± 3.2	Δ −5.0 ± 3.3	Δ −6.8 ± 3.7	
Tongue coating score (0–3)	Baseline 1.7 ± 0.7	Baseline 1.7 ± 0.7	Baseline 1.7 ± 0.7	<0.001
	Week 4 1.4 ± 0.8	Week 4 1.2 ± 0.8	Week 4 1.0 ± 0.7	
	Δ −0.3 ± 0.4	Δ −0.5 ± 0.4	Δ −0.7 ± 0.4	
Plaque index (0–3)	Baseline 1.6 ± 0.5	Baseline 1.6 ± 0.6	Baseline 1.7 ± 0.6	0.033
	Week 4 1.5 ± 0.5	Week 4 1.4 ± 0.6	Week 4 1.4 ± 0.6	
	Δ −0.1 ± 0.3	Δ −0.2 ± 0.3	Δ −0.3 ± 0.3	
*So. moorei* (log10 copies/mL)	Baseline 6.4 ± 0.6	Baseline 6.4 ± 0.6	Baseline 6.4 ± 0.6	<0.001
	Week 4 6.2 ± 0.7	Week 4 5.8 ± 0.7	Week 4 5.7 ± 0.7	
	Δ −0.2 ± 0.5	Δ −0.6 ± 0.5	Δ −0.7 ± 0.5	
Moderate/severe halitosis at week 4 (organoleptic ≥ 2.5)	23 (58.9)	18 (46.2)	8 (20.5)	0.004

Data are shown as mean ± SD or n (%). Δ, change from baseline to week 4; VSCs, volatile sulfur compounds; ppb, parts per billion; HALT, Halitosis Associated Life-Quality Test (0–100); OHIP-14, Oral Health Impact Profile-14 (0–56); *So. moorei*, *Solobacterium moorei*.

**Table 6:** Spearman correlation matrix at baseline.

Variable	VSC	Organoleptic	HALT	OHIP-14	Plaque	Tongue Coat	Salivary Flow	*So. moorei*	Reflux Score
VSC	—	0.7 ***	0.4 ***	0.3 ***	0.3 ***	0.5 ***	−0.4 ***	0.3 ***	0.2 *
Organoleptic	0.7 ***	—	0.5 ***	0.4 ***	0.3 ***	0.4 ***	−0.4 ***	0.3 ***	0.2 *
HALT	0.4 ***	0.5 ***	—	0.5 ***	0.1	0.3 ***	−0.2 *	0.2 *	0.3 ***
OHIP-14	0.3 ***	0.4 ***	0.5 ***	—	0.1	0.2 *	−0.1	0.1	0.2 *
Plaque	0.3 ***	0.3 ***	0.1	0.1	—	0.2 *	−0.1	0.1	0.1
Tongue coat	0.5 ***	0.4 ***	0.3 ***	0.2 *	0.2 *	—	−0.2 *	0.4 ***	0.2 *
Salivary flow	−0.4 ***	−0.4 ***	−0.2 *	−0.1	−0.1	−0.2 *	—	−0.1	−0.1
*So. moorei*	0.3 ***	0.3 ***	0.2 *	0.1	0.1	0.4 ***	−0.1	—	0.1
Reflux score	0.2 *	0.2 *	0.3 ***	0.2 *	0.1	0.2 *	−0.1	0.1	—

Spearman ρ coefficients shown. VSC, volatile sulfur compounds; HALT, Halitosis Associated Life-Quality Test; OHIP-14, Oral Health Impact Profile-14; *So. moorei*, *Solobacterium moorei*. * *p* < 0.05; *** *p* < 0.001.

**Table 7:** Multivariable logistic regression predicting moderate/severe halitosis at week 4 (organoleptic ≥ 2.5).

Predictor	OR (95% CI)	*p* Value
Probiotic lozenge vs. standard care	0.44 (0.20–0.96)	0.039
Zinc mouthrinse vs. standard care	0.13 (0.05–0.33)	<0.001
Low salivary flow (<0.25 mL/min)	2.74 (1.20–6.24)	0.017
High reflux symptoms (score ≥ 6)	3.62 (1.54–8.52)	0.003
Baseline organoleptic > 3.0	2.05 (0.94–4.47)	0.073
Tongue coating ≥ 2.0	1.62 (0.76–3.43)	0.213

Model discrimination (exploratory): area under curve (AUC) = 0.78. OR, odds ratio; CI, confidence interval.

**Table 8:** Subgroup analysis by salivary flow: change scores (Δ) by arm.

Salivary Flow Stratum	Arm	n	Δ VSC (ppb)	Δ Organoleptic	Δ HALT
Low flow	Standard care	11	6.3 ± 33.7	−0.1 ± 0.7	−6.2 ± 7.6
Low flow	Probiotic lozenge	10	−25.2 ± 37.5	−0.6 ± 0.7	−10.7 ± 9.6
Low flow	Zinc mouthrinse	10	−57.4 ± 32.8	−0.9 ± 0.7	−15.2 ± 9.6
Low flow	*p* (between arms, ANCOVA)	0.041	0.017	0.075	
Normal flow	Standard care	28	−20.2 ± 33.2	−0.2 ± 0.7	−11.4 ± 7.0
Normal flow	Probiotic lozenge	29	−55.1 ± 42.7	−0.9 ± 0.8	−17.5 ± 7.0
Normal flow	Zinc mouthrinse	29	−85.7 ± 34.0	−1.3 ± 0.8	−22.8 ± 7.0
Normal flow	*p* (between arms, ANCOVA)	<0.001	<0.001	<0.001	
Interaction p (arm × flow)		0.428	0.946	0.654	

Data are shown as mean ± SD. Δ, change from baseline to week 4; VSCs, volatile sulfur compounds; ppb, parts per billion; HALT, Halitosis Associated Life-Quality Test (0–100); ANCOVA, analysis of covariance. Low salivary flow defined as unstimulated flow < 0.25 mL/min.

**Table 9:** Pairwise differences in mean change.

Outcome	Probiotic − Standard (Δdiff)	Zinc − Standard (Δdiff)	Zinc − Probiotic (Δdiff)
VSC (ppb)	−34.6 (95% CI −51.8 to −17.4)	−65.8 (95% CI −80.5 to −51.1)	−31.2 (95% CI −47.3 to −15.1)
Organoleptic	−0.6 (−0.9 to −0.3)	−1.0 (−1.3 to −0.7)	−0.4 (−0.7 to −0.1)
HALT	−5.8 (−9.4 to −2.2)	−11.0 (−14.6 to −7.4)	−5.2 (−8.8 to −1.6)
OHIP-14	−2.1 (−3.6 to −0.6)	−3.9 (−5.5 to −2.3)	−1.8 (−3.4 to −0.2)
Tongue coating	−0.2 (−0.4 to 0.0)	−0.4 (−0.6 to −0.2)	−0.2 (−0.4 to 0.0)
Plaque index	−0.1 (−0.2 to 0.0)	−0.2 (−0.3 to −0.1)	−0.1 (−0.2 to 0.0)
*So. moorei* (log10)	−0.4 (−0.6 to −0.2)	−0.5 (−0.7 to −0.3)	−0.1 (−0.3 to 0.1)

Interpretation: negative values indicate greater improvement/reduction vs. comparator.

## Data Availability

The data presented in this study are available on request from the corresponding author.
